# Study on Bioresponsive Gelatin-Hyaluronic Acid-Genipin Hydrogel for High Cell-Density 3D Bioprinting

**DOI:** 10.3390/gels9080601

**Published:** 2023-07-26

**Authors:** Mst Rita Khatun, Amitava Bhattacharyya, Maral Gunbayar, Minsik Jung, Insup Noh

**Affiliations:** 1 Department of Chemical and Biomolecular Engineering, Seoul National University of Science and Technology, Seoul 01811, Republic of Korea; mstrita931215@seoultech.ac.kr (M.R.K.); amitbha1912@seoultech.ac.kr (A.B.); marlagunbayer@naver.com (M.G.); minsik0217@naver.com (M.J.); 2 Functional, Innovative and Smart Textiles, PSG Institute of Advanced Studies, Coimbatore 641004, India; 3 Convergence Institute of Biomedical Engineering and Biomaterials, Seoul National University of Science and Technology, Seoul 01811, Republic of Korea

**Keywords:** bioresponsive, gelatin, hyaluronic acid, bioprinting, cell-delivery, tissue engineering

## Abstract

The Development of bioresponsive extrudable hydrogels for 3D bioprinting is imperative to address the growing demand for scaffold design as well as efficient and reliable methods of tissue engineering and regenerative medicine. This study proposed genipin (5 mg) cross-linked gelatin (1 to 1.5 g)-hyaluronic acid (0.3 g) hydrogel bioink (20 mL) tailored for 3D bioprinting. The focus is on high cell loading and a less artificial extra-cellular matrix (ECM) effect, as well as exploring their potential applications in tissue engineering. The bioresponsiveness of these hydrogel scaffolds was successfully evaluated at 37 °C and room temperature (at pH 2.5, 7.4, and 9). The rheological and mechanical properties (more than three times) increased with the increase in gelatin content in the hydrogel; however, the hydrogel with the least amount of gelatin showed the best extrusion capability. This optimized hydrogel’s high extrusion ability and post-printing shape fidelity were evident from 3D and four-axis printing of complex structures such as hollow tubes, stars, pyramids, and zigzag porous tubular (four-axis) scaffolds (printed at 90 kPa pressure, 70 mm/s speed, 22G needle, fourth axis rotation of 4 rpm). 3 million/mL MC3T3-E1 mouse osteoblast cells were used in preparing 3D bioprinted samples. The in vitro cell culture studies have been carried out in a CO_2_ incubator (at 37 °C, 5% CO_2_). In the cytocompatibility study, almost three times more cell viability was observed in 3 days compared to day 1 control, proving the non-toxicity and cell-supportiveness of these hydrogels. High cell viability and cell-to-cell interactions observed at the end of day 3 using this moderately stable hydrogel in 3D bioprinting exhibit high potential for precise cell delivery modes in tissue engineering as well as regenerative medicine.

## 1. Introduction

3D Bioprinting is widely used in tissue engineering and regenerative medicine for fabricating complex artificial tissue and organ structures that can mimic natural tissues and organs [1]. As the success of tissue formation depends on the nature of bioinks used in 3D bioprinting, various polymeric hydrogels have been studied in response to the increasing significance of 3D bioprinting and injectable hydrogels in these fields [2]. The physical, chemical, and biological properties of polymeric hydrogels vary widely depending on their biomedical application areas, including tissue engineering scaffolds, biosensors, burn dressings, drug delivery, cell encapsulation, and bioprinting. Most biodegradable hydrogels for tissue engineering scaffolds and biosensors may not be suitable for bioprinting, requiring a better understanding of the advantages and weaknesses of the fabricated hydrogels for various applications [3].

A new class of hydrogel, named bioresponsive hydrogels, has gained researchers’ interest in diagnostics, drug delivery, tissue regeneration, and wound healing [4]. Bioresponsive hydrogels respond to external stimuli or stimulate specific biological signals through natural biological processes [5]. These bioresponsive hydrogels change their physico-chemical and biological properties spatio-temporally. They swell, collapse, or degrade over time in response to selective biological environments [6]. Bioresponsive hydrogels are under extensive research for targeted cell delivery, cancer therapy, drug delivery, tissue engineering, and 3D bioprinting systems with and without scaffolds [7,8,9]. Scaffold-based and scaffold-free 3D cell culture systems have been developed to establish related in vitro models replicating the complex structure observed in vivo [10]. Scaffold-based cell-embedded 3D bioprinting systems often suffer from a limitation of less cell interaction and communication as well as difficulty matching the time for scaffold degradation and tissue regeneration, especially in slow degradation biomaterials [11]. This can be addressed using high cell density 3D bioprinting of hydrogels and controlled degradation of the biomaterials to generate better cell-cell interactions, which are important for the functioning of the artificial neotissue. High cell density and a smaller amount of hydrogel minimize the effect of artificial extracellular matrix, leading to a close replica of the natural tissues [12].

Several natural biomaterials are used in fabricating bioresponsive extrudable hydrogels for 3D bioprinting. Gelatin, derived from denatured collagen, is a commonly employed biopolymer for hydrogel formation, finding wide application in pharmaceuticals, food, medical, and tissue engineering fields [13]. Gelatin-based hydrogels possess several favorable characteristics, including biocompatibility, non-toxicity, non-immunogenicity by using atelocollagen, biodegradability, cost-effectiveness, and easy accessibility. These qualities make them highly suitable materials for various biomedical applications [14]. However, the gel formed through the physical crosslinking of gelatin lacks structural stability and quickly dissolves when exposed to high temperatures [15]. The mechanical and physical properties of gelatin hydrogel can be improved by the addition of other polymers and chemical crosslinking. Hyaluronic acid (HA), also known as hyaluronan, is a vital biomaterial used in biocompatible gels. It is an anionic, non-sulfated glycosaminoglycan comprising repeating units of D-glucuronic acid and N-acetyl-D-glucosamine. HA is present in various tissues, including connective, epithelial, and neural tissues [16,17,18,19]. In recent years, researchers have been modifying HA by incorporating natural polymers, synthetic polymers, or nanoparticles. These modifications are used to develop composite materials, hydrogels, or hydrogel nanocomposites [20,21]. Genipin has been employed as a strong yet safe cross-linker of proteins like collagen, gelatin, and polysaccharides such as chitosan. This is preferred for crosslinking bioink polymers during 3D bioprinting as it possesses some anti-inflammatory qualities [14].

**Figure 1:**
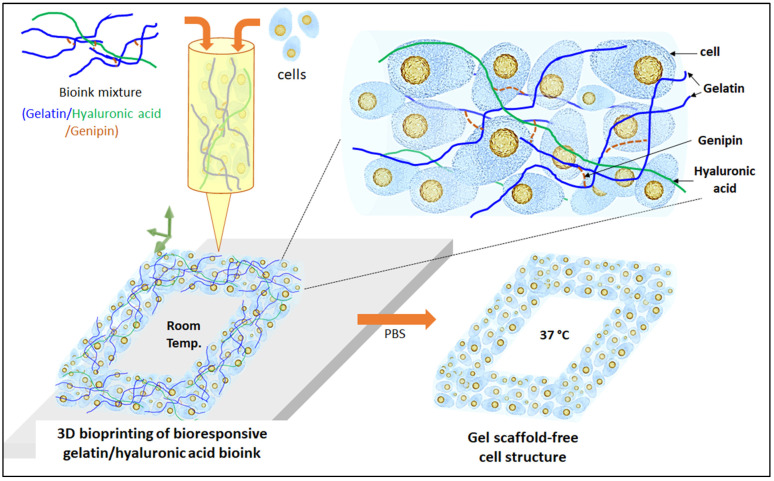
Scheme of 3D bioprinting with bioresponsive gelatin-hyaluronic acid hydrogel.

In this present study, gelatin-HA bioresponsive hydrogel is cross-linked with genipin for 3D bioprinting of tissue engineering scaffolds with high shape fidelity and resolution. Here, positively charged gelatin is used to improve the polyelectrolyte complex formation with negatively charged HA. Genipin crosslinking with gelatin polymers gives the hydrogel further stability and extrudable properties. This hydrogel is expected to be stable at room temperature, while its stability (bioresponsiveness) needs to be assessed under physiological conditions such as body temperature and pH. Three different amounts of gelatin were used to prepare gelatin-HA hydrogel in a fixed amount of HA (1 g, 1.2 g, and 1.5 g gelatin, each with 0.3 g HA in 20 mL distilled water, coded as Gel 1, Gel 2, and Gel 3, respectively), cross-linked with genipin (5 mg). Figure 1 shows the present study scheme for 3D bioprinting with this extrudable gelatin-HA acid hydrogel. Analysis of the hydrogel’s rheological and mechanical properties, high-resolution 3D printing as well as four-axis printing, and cytocompatibility studies are carried out using this bioresponsive hydrogel. High cell density is used in the gelatin-HA hydrogel for 3D bioprinting. During and after the degradation of this hydrogel scaffold, cell-to-cell interaction would increase in this 3D bioprinting system, leading to better functional tissue formation by minimizing scaffold effects.

## 2. Results and Discussion

### 2.1. Gelatin-HA Hydrogel Formation and It’s Bioresponsiveness

**Figure 2:**
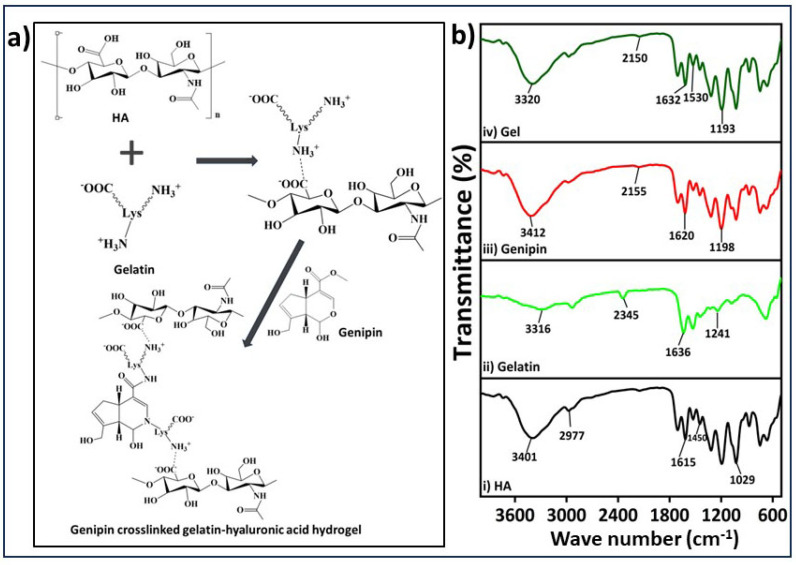
(**a**) Proposed reaction mechanism of the gelatin-HA gel formation with genipin, (**b**) FTIR analysis of hyaluronic acid (HA); gelatin, genipin, and gel (**i**, **ii**, **iii**, **iv** respectively).

The process of gelatin-HA hydrogel formation occurs sequentially in two stages: first, polyelectrolyte complexing, and then genipin crosslinking with gelatin, as proposed in Figure 2a. The bonding of gelatin with negatively charged HA through polyelectrolyte complex formation is better initiated at a lower pH. Below the isoelectric point, gelatin is positively charged, and the bond formation with HA is expected to be more stable. In the cross-linking process using genipin, the amine group’s free electrons in the gelatin polymer initiate a nucleophilic attack on the nearby hydroxyl group of genipin’s ether group with unsaturation, leading to the opening of the cyclic structure and the formation of an intermediate aldehyde group (Figure 2a). This intermediate aldehyde group then reacts with the amine group, forming an intermediate nitrogen-containing ring structure. Furthermore, the free electron on the gelatin amine group reacts with the ester group at the upper end of genipin, resulting in the formation of an amide bond. This amide bond contributes to both intramolecular and intermolecular cross-linking of the gelatin network. The FTIR spectra depicted in Figure 2b exhibit distinct peaks corresponding to hyaluronic acid (HA). These peaks occur at wavelengths of 3401, 1615, and 1450, representing the stretching vibrations of the O-H or N-H group, the C=O group, and the C-O bonds of the -COO- group, respectively [22]. The presence of water (O-H stretching) and amide A resulted in a band at 3316 cm^−1^ in the FTIR of gelatin’s chemical structure (Figure 2(bii)). The presence of amide-I in gelatin was confirmed by the peak at 1636 cm^−1^ [23]. The FTIR analysis of genipin revealed specific absorption peaks at different wavelengths. One peak at 1680 cm^−1^ was associated with the stretching vibrations of the carboxymethyl carbonyl group (C=O), while another peak at 1620 cm^−1^ was related to the C=C vibration of the olefin ring in genipin. The presence of a double peak at 3412 cm^−1^ in the genipin spectrum was likely due to the overlapping of vibrations from aromatic carbon-hydrogen (C–H) bonds and hydroxyl (O–H) groups [24]. The FTIR spectrum of hydrogel demonstrates the presence of the cross-linking amide bond (-CONH-) at approximately 1530 and 1632 cm^−1^, confirming the strong association between the gelatin and genipin, which aligns with the previously described reaction mechanism in Figure 2a.

**Figure 3:**
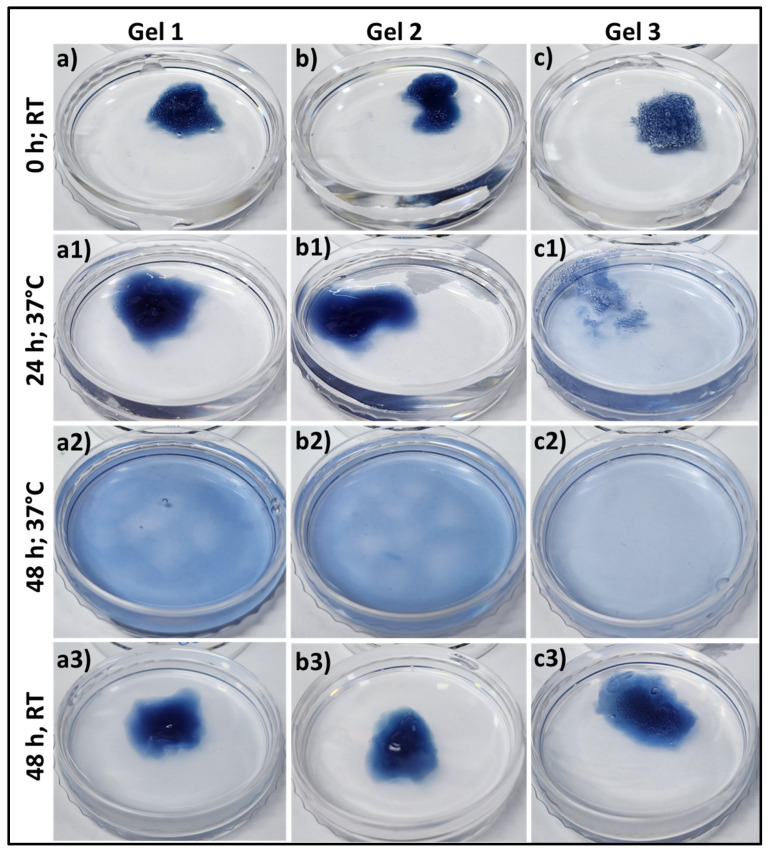
Swelling & degradation study of different gelatin-HA hydrogels at 37 °C and room temperature (25 °C) into phosphate buffer, pH 7.4 for 48 h. (**a**–**c**,**a1**–**c1**,**a2**–**c2**) at 37 °C; (**a3**–**c3**) at room temperature after 48 h.

The bioresponsiveness of these three different gelatin-HA hydrogels has been studied at different temperatures and times. Figure 3 represents the swelling and dissolving of the different compositions of gels in phosphate buffered saline, pH 7.4, at 37 °C and room temperature. From Supporting Figure S1, all these three different gelatin-HA hydrogels swelled for the first 6 h due to the gelling property of these cross-linked hydrogels. After that, the gels started disintegrating, and within 48 h, Gel 3 was completely dissolved. After 48 h, Gel 1 and Gel 2 have some portions remaining after disintegration. Other than gelatin, the amounts of all components, including cross-linker genipin, are constant in all hydrogel compositions. The increased amount of gelatin reduces the crosslinking sites of the hydrogel, making it more vulnerable to temperature and ion-induced disintegration. At room temperature, all three compositions of gel didn’t dissolve away after 48 h. Supporting Figure S2 shows the swelling and dissolving of the three compositions of hydrogel at different pHs (pH 2.5 and pH 9) at 37 °C. There was no swelling or dissolving observed in all three compositions of hydrogel after 48 h in acidic media, and Gel 1 and Gel 2 remained stable after 5 days (Supporting Figure S3).

In alkaline media, all the gels swelled first, and Gel 3 completely dissolved within 48 h. Gel 2 almost dissolved away, and Gel 1 dissolved very little. Strong polyelectrolyte bonding between gelatin and HA may justify the very low dissolution under acidic conditions. Gelatin provides more positive charge in acidic buffer media because of its high isoelectric point, which makes the bond stronger with negatively charged HA. Genipin crosslinking with gelatin formed a moderately stable and extrudable gelatin-HA hydrogel by overcoming the limitations of its lower stability [15]. These data successfully prove the bioresponsiveness of these hydrogels. Hydrogels with a short duration of stability typically dissolve rapidly over time, which can be problematic for long-term tissue engineering applications. The rapid dissolution compromised the structural integrity and functionality of printed structures. Despite these limitations, this kind of moderately stable hydrogel offers an environment that supports high cell viability during and after the 3D bioprinting process. The gentle and controlled printing conditions associated with these hydrogels minimize cell damage and maintain cell functionality. Additionally, the short-term stability of these hydrogels allows for their subsequent removal or dissolution, reducing any potential negative effects on the encapsulated cells. These hydrogels can be applied for high cell-density bioprinting, cell delivery applications, drug delivery applications, and functional tissue formation by minimizing the scaffold effect.

### 2.2. Rheological and Mechanical Properties of the Gelatin-HA Hydrogels

**Figure 4:**
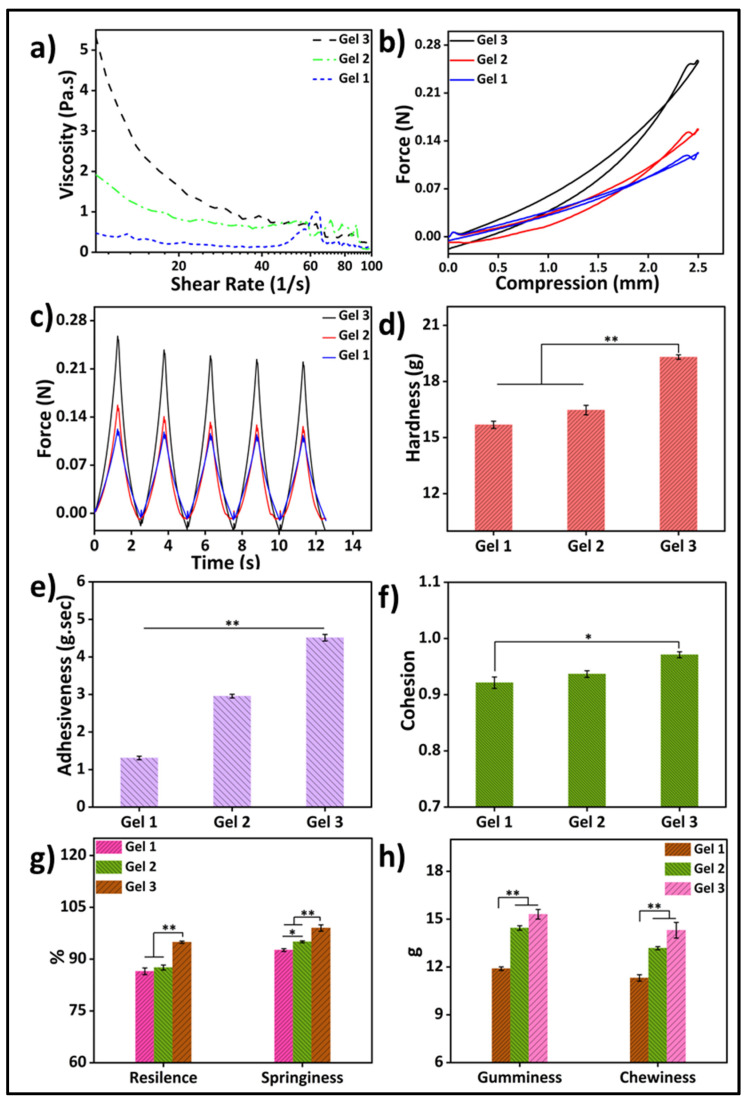
(**a**) Rheological properties of three different gelatin-HA hydrogels; Cyclic compressive response of the hydrogels (**b**) with compression; (**c**) with time; Mechanical properties of three different hydrogels (**d**) Hardness; (**e**) Adhesiveness; (**f**) Cohesion; (**g**) Resilience & Springiness; (**h**) Gumminess & Chewiness. Results differences are considered statistically significant when *p* ≤ 0.05 (*) and *p* ≤ 0.01 (**).

Rheological property analysis of gelatin-HA hydrogels involves the study of their flow and deformation characteristics under various conditions. It plays an important role in understanding the behavior of hydrogels, which are composed mostly of water. Viscosity is an important rheological property that refers to the resistance of a material to flow. It is an essential property to study in hydrogels as it determines their ability to flow or deform under applied stress. Figure 4a depicts the rheological property analysis of the three different compositions of the hydrogels. Parallel-plate rotating discs were used to study the rheological behavior of bioink. This study method is recognized by the researchers as necessary to comprehend the rheology of bioink for extrusion-based 3D bioprinting [25]. The viscosity of the gels increased as the gelatin concentration increased, and all the samples showed shear-thinning behavior. The viscosities of Gel 1, Gel 2, and Gel 3 were 0.303 Pa.s, 1.026 Pa.s, and 2.18137 Pa.s, respectively, at a shear rate of 10 s^−1^. The shear-thinning property of gel is advantageous for its application in bioprinting, as it enables high-resolution printing and ensures structural integrity.

A texture analyzer was used to study the cyclic compressive mechanical properties (Figure 4b–h). The compressive strengths and other mechanical properties of the gel samples were significantly higher with the higher gelatin content in the samples. The strength at 25% compression increased four times more in 1.5 g of gelatin containing Gel 3 than in 1 g of gelatin containing Gel 1. Again, hardness (Gel 1: 15.68 g, Gel 2: 16.47 g, and Gel 3: 19.3 g) increased with the increase in gelatin amount. Similarly, adhesiveness, cohesion, resilience, springiness, and gumminess were also increased with the increase in gelatin content in the hydrogels. This may be because of the higher mass content in the hydrogel with increasing amounts of gelatin. To achieve enhanced stability and improve the physical, mechanical, and biological properties of hydrogels, researchers have found that increasing the amount of gelatin in the hydrogel formulation is effective. This increase in gelatin quantity has been associated with improved mechanical strength and increased adhesiveness, evident from a more pronounced negative load region during retraction. The hardness of the gel reflects its strength, while gumminess and chewiness represent its textural properties during crunching. Adhesiveness refers to the gel’s ability to adhere to other surfaces, while cohesiveness indicates its strength in maintaining its own structure. Springiness is the hydrogel’s capacity to regain its original shape after deformation, while resilience measures the energy loss during such deformation [25]. Controlling these properties, including hardness, cohesiveness, adhesiveness, chewiness, and gumminess, is crucial for hydrogels to enhance printability and mechanical properties in 3D bioprinting applications. Several studies have highlighted the use of higher amounts of gelatin to achieve these improvements.

### 2.3. Morphology of the Gelatin-HA Hydrogels

**Figure 5:**
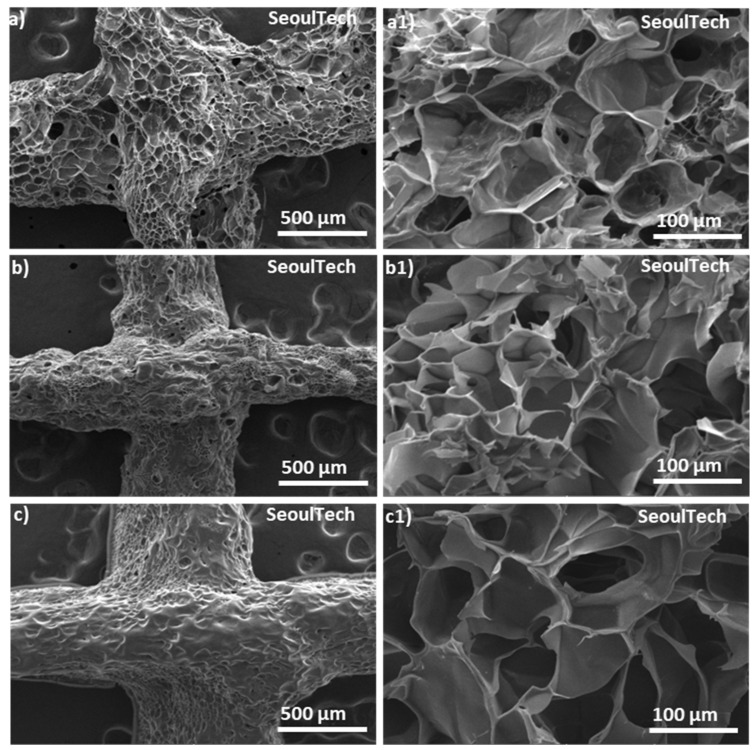
SEM images of the surface and cross-section of the three different gelatin-HA hydrogels. Surface and cross-section images of Gel 1 (**a,a1**), Gel 2 (**b**,**b1**), and Gel 3 (**c**,**c1**), respectively.

Scanning electron spectroscopy (SEM) analysis allows for high-resolution imaging of the hydrogel surface, providing information about its morphology, roughness, and surface features. It helps to observe the microstructure of the gelatin-HA hydrogel, such as the presence of pores, surface irregularities, or surface modifications. Surface morphology is very important for applications in tissue engineering, where surface characteristics influence cell adhesion and growth. Again, from the SEM of the cross-sections, the internal structure of the hydrogel, including pore distribution, interconnectivity, and homogeneity, can be analyzed. This information is crucial for understanding the transport properties of the hydrogel, such as the diffusion of nutrients, drugs, or bioactive molecules within the hydrogel. SEM analysis was carried out to check the structural changes of the three different compositions of gel with the increase in gelatin content. In Figure 5, SEM images revealed the porous surface and cross-section structure of the three different gelatin-HA hydrogels. SEM images clearly showed that the porosity sharply decreased with the increase in gelatin amount in the hydrogel, which proves the structural compactness of the high gelatin-containing hydrogel. Gel 1 showed a higher porous surface and cross section morphology of the hydrogel than Gel 2 and Gel 3. The uniform pore distribution and higher porous structure prove the hydrogel’s well-defined and fully cross-linked gel network. Again, with the increase in gelatin amount in the hydrogel, the porosity decreased and the pore size increased, which proves the lower crosslinking density in the hydrogel. From the figure, Gel 1 is fully cross-linked, and after that, the genipin amount was insufficient to crosslink the higher amount of gelatin, which is responsible for the less irregular surface and cross-section morphology of the hydrogel. Porous morphology and a uniform cross-linked surface are very important for cell adhesion as well as drug encapsulation in ideal biomaterial-based hydrogel scaffolds [20].

### 2.4. 3D Printability of the Gelatin-HA Hydrogels for Complex Structures

**Figure 6:**
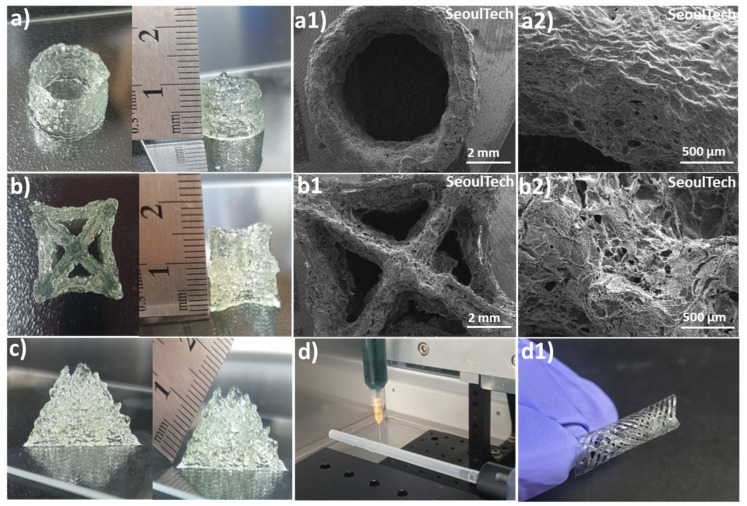
3D and four-axis printing of complex structures with gelatin-HA gel. (**a**,**a1**,**a2**) Digital and SEM images of Hollow tube; (**b**,**b1**,**b2**) Digital and SEM images of Star shape; (**c**) Pyramid shape; (**d**,**d1**) Four-axis printed tubular shape.

The hydrogels exhibit a shear-thinning property, which means that they can easily flow and be extruded during the extrusion-based 3D printing process. The parameters such as printing speed, air pressure, and printing diameter were optimized first to ensure optimal printing conditions. After analysis of physical properties and extrusion ability, Gel 1 was optimized for 3D printing as well as four-axis printing. Using these optimized parameters, various shapes were successfully printed, including hollow tubes and star shapes, each composed of approximately 50 layers and reaching a height of 1 cm (Figure 6a,b). Upon lyophilization, SEM images (Figure 6(a1,a2,b1,b2)) revealed a porous surface morphology in these complex printed shapes. The 3D printed structures made from gelatin-HA gels, particularly a pyramid-like structure (Figure 6c, 1.5 cm height and around 100 layers), represented outstanding stability and maintained their shape fidelity after printing. An interesting observation was that the printed structures firmly adhered to the substrates without the need for any additional adhesive, representing the high adhesiveness of the hydrogel to the surface. Even after inverting the printed scaffolds multiple times with the surface, the structures remained intact and didn’t detach (Supporting Figure S4). In brief, the hydrogels with shear-thinning properties demonstrated outstanding extrusion ability and printability on an extrusion-based 3D printer. The optimized parameters allowed the successful printing of different shapes, and the resulting printed structures exhibited a porous morphology. Notably, the printed structures exhibited remarkable stability, maintained their shape fidelity, and firmly adhered to glass substrates without additional adhesives, indicating the high adhesiveness of the hydrogel. The robustness of the printed structures was evident, as they remained intact even after repeated inversions with the substrate.

The hydrogel was successfully used for four-axis printing, as shown in Figure 6(d,d1). This printing method uses a spinning plastic tube connected to a speed controller as a base for printing. By editing G-codes, the rod’s movement in the X and Y axes was controlled while maintaining a constant syringe height. The G-codes were adjusted to achieve a porous structure in the tubular structures. During the printing process, the plastic tube moved horizontally backward and forward, as demonstrated in Supporting Video S1. A crucial aspect to consider here is the high adhesive property of the gel during the rotation of the plastic tube substrate during 3D printing. For successful printing, the gel must have both self-cohesiveness and adhesion to the plastic surface. This allows it to form a tubular shape that can resist detaching from the hydrophobic, inert surface. If the gel lacks sufficient cohesion and adhesion, the printed layers become unstable, and there is a risk of the structure collapsing when the plastic tube rotates. The cohesive and adhesive properties of the hydrogel were essential in maintaining the stability of the printed layers and structures.

After printing, the scaffolds were removed from the tube after air drying for a few minutes. The samples showed very high shape retention and stability. The flexibility, such as stretching, bending, expansion, etc., of the printed constructs can be observed in Supporting Video File S2. Additionally, by adjusting the synthesis parameters and compositions of the bioink, the resolution of the printed structures can be modified or scaled to suit specific applications. This approach can also be extended to the development of nerve conduits using different biomaterials, bone tissue engineering scaffolds, or stents. The experiments effectively show that bioink gels can efficiently print complex and self-supporting large structures in a short time with consistent results.

### 2.5. Cell Culture Studies on 3D Bioprinted Gelatin-HA Hydrogels

**Figure 7:**
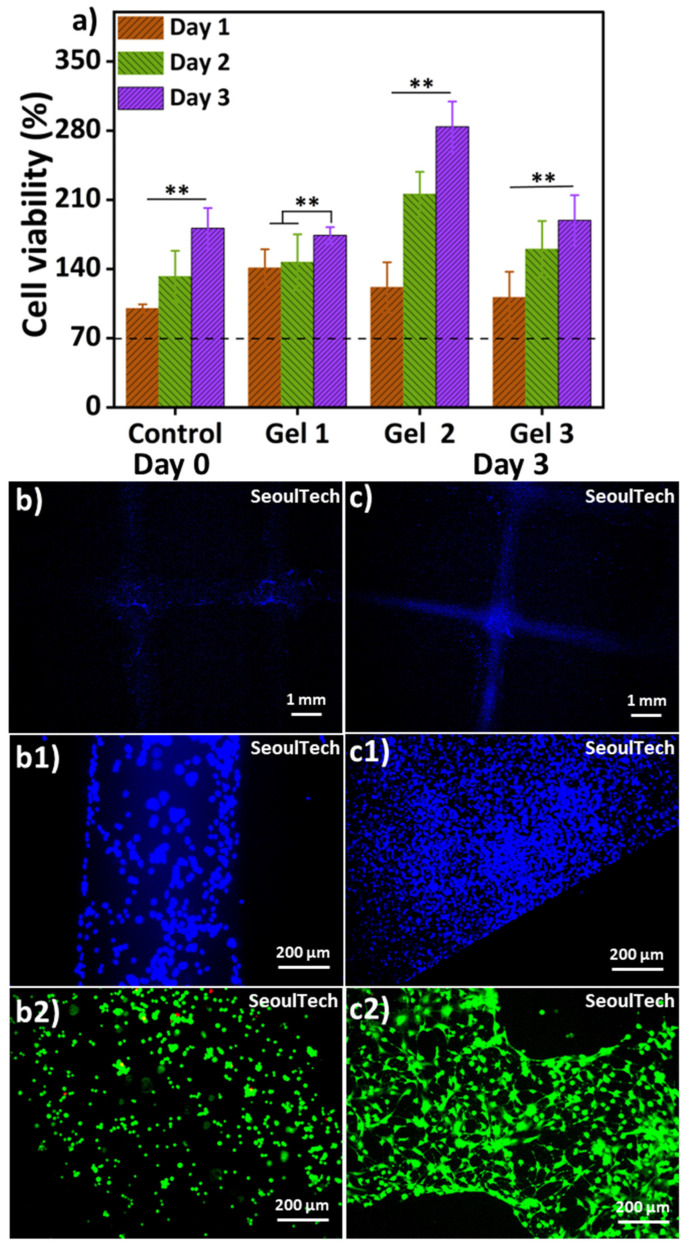
(**a**) MTT assay of three different conditions hydrogel; (**b**,**c**) DAPI staining low magnification images at day 0 and day3; (**b1**,**c1**) DAPI staining high magnification images at day 0 and day 3; (**b2**,**c2**) Live and Dead (L&D) staining using Max-view^TM^ Live/Dead cell staining kit (Biomax, Guri, Republic of Korea) high magnification images at day 0 & day 3, where green represents live cells and red represents dead cells. Results differences are considered statistically significant when *p* ≤ 0.01 (**).

Figure 7 presents the results of an in vitro cell study using mouse osteoblast MC3T3 cells. Genipin is a strong but safe substance that can bind proteins like chitosan, collagen, and gelatin together. Recent studies have shown that when genipin is added to these materials, it gives them anti-inflammatory properties [26]. Genipin was suggested long ago as a substitute for glutaraldehyde, a fixative for biological tissues [27]. It was proven to be significantly less toxic, about 10,000 times, while being nearly as effective as glutaraldehyde. The MTT assay (Figure 7a) was performed for up to 3 days, and all samples exhibited cell viability exceeding 70%, indicating that the hydrogels used in this study are supportive of cell growth and non-toxic [15,22]. The MTT assay involved extracting substances from the cell-encapsulated hydrogels for analysis. The cell viability increased over time in all samples, suggesting that the hydrogels are non-toxic. The bioink used in this study for crosslinking the biocompatible components did not contain any toxic substances.

**Figure 8:**
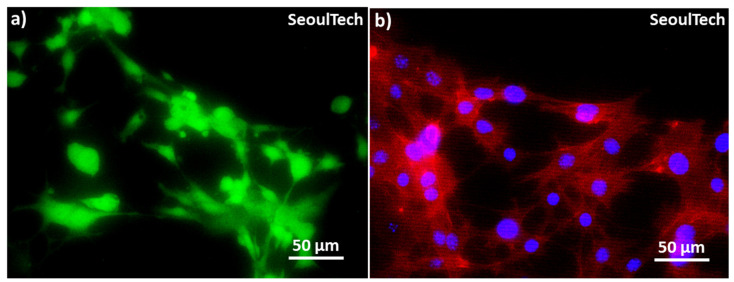
High magnification Live and Dead and DAPI/actin images at the end of day 3. (**a**) L&D image & (**b**) DAPI/actin image, where blue represents nucleus and red represents actin filaments.

Figure 7(b,b1,c,c1) showed the DAPI (4′,6-diamidino-2-phenylindole) stained images of the blue nucleus at day 0 and day 3. The high cell-density 3D bioprinted grid-like structure with 3 million/mL MC3T3-E1 cells is preserved after disintegration of the hydrogel components, as shown in the low magnification Day 3 DAPI image (Figure 7c). The cells covered the entire structure. The day 3 live and dead (L&D) result reveals cell-to-cell interaction/communication after the complete disintegration of ECM (Figure 7(c2)). Furthermore, the cytoskeletons of the cells exhibit a spreading morphology and improved structural strength after three days of culture. The live/dead results (Figure 7(b2)) at Day 0 showed that the live cells were evenly distributed within the gel matrix, where green represents live cells and red represents dead cells. The three-day study further confirmed that these hydrogels are supportive of cell growth. The high cell-density 3D bioprinted structure demonstrated consistent cell-delivery for scaffold formation upon the complete disintegration of the ECM. Within three days (Figure 7(c2)), the gel matrix completely disintegrated, the cells multiplied, and cell-to-cell interaction and communication increased, forming scaffold-shaped tissue. High magnification images of L&D and DAPI/actin (red rhodamine-phalloidin for F-actin) from Figure 8 clearly show the excellent cell-to-cell interaction and communication established after disintegration of the hydrogel. This has the possibility of loading fully functional tissue formation using this method of high cell-density 3D bioprinting with the studied gelatin-HA hydrogel.

## 3. Conclusions

In this study, a bioresponsive gelatin-hyaluronic acid hydrogel cross-linked with genipin has been developed for high cell-density 3D bioprinting. Here, positively charged gelatin was bonded with negatively charged hyaluronic acid through polyelectrolyte complex formation. Furthermore, the free electron on the gelatin amine group reacts with the ester group at the upper end of genipin, resulting in the formation of an amide bond. This amide bond contributes to both intramolecular and intermolecular cross-linking. The bioresponsiveness of this hydrogel was evaluated under physiological conditions. These hydrogels disintegrated within 48 h at 37 °C to pH 7.4 in the presence of ions in phosphate buffer saline, whereas they remained stable at room temperature. Again, the gels disintegrated at pH 9 within 24 h but remained stable at pH 2.5, which proves the strong polyelectrolyte bond between gelatin and hyaluronic acid during gel formation in acidic or neutral conditions. The rheological and mechanical properties show that with the increase in gelatin content in the hydrogel, the properties increased gradually. SEM analysis showed the porous morphology of the hydrogels. Successful 3D printing and four-axis printing prove that the optimized composition of hydrogel possesses excellent extrusion ability and shape-fidelity properties. An in vitro cell study showed the non-cytotoxicity and high cell supportiveness of this hydrogel. High cell-density 3D bioprinting has been carried out successfully, and upon the hydrogel scaffold’s disintegration, the natural ECM effects on cell-cell communications. High cell-to-cell interaction and communication and the formation of scaffold-shaped tissue would have a high potential for the application of this hydrogel in regenerative medicine in areas such as cartilage, skin, blood vessels, and others.

## 4. Materials and Methods

### 4.1. Materials

Hyaluronic acid (Kindly donated by Ildong Pharmaceutical company (AK0701 batch, Seoul, Republic of Korea); Gelatin from porcine skin (gel strength 300); and Genipin (MW: 226.23) were purchased from Sigma Aldrich (St. Louis, MO, USA). Mouse Osteoblast cells (Passage 11, MC3T3-E1 cell line) were purchased from Young Science Inc. (Bucheon, Republic of Korea). α-Minimum essential media (MEM) purchased from Sigma Aldrich (St. Louis, MO, USA); FBS (10% fetal bovine serum) purchased from Gibco (ThermoFisher Scientific Korea, Seoul, Republic of Korea); penicillin-streptomycin purchased from Sigma Aldrich, St. Louis, MO, USA; Max-viewTM Live/Dead cell staining kit purchased from Biomax (Guri, Republic of Korea); 4′,6-diamidino-2-phenylindole (DAPI) and rhodamine phalloidin (F-actin) staining kit purchased from Thermo Fisher Scientific Korea (Seoul, Republic of Korea).

### 4.2. Synthesis of Hydrogel

0.3 g of hyaluronic acid was dissolved into 10 mL of distilled water (DW) by stirring overnight at 400 rpm. Different amounts of gelatin (1 g, 1.2 g, and 1.5 g) were dissolved into 8 mL of DW and stirred for 1 h. 5 mg of genipin was dissolved separately into 2 mL of DW. The gelatin solution was mixed with the hyaluronic acid solution while stirring for 1 h. After 1 h, the genipin solution was mixed with the HA-gelatin solution and stirred for 30 min. After 30 min, the resulting product was kept at room temperature for 3 h for gelation. Table 1 shows the composition of the hydrogels and the codes used in this study.

### 4.3. Characterization of Gelatin-HA Hydrogel

A Varian 640-IR spectrometer from ALT (San Diego, CA, USA), was used to conduct Fourier transform infrared spectroscopy (FTIR) on lyophilized gels and raw materials. The wavelength range analyzed was 400–4000 cm^−1^. Rheological properties of the hydrogels were evaluated using an R/S Plus rheometer (Brookfield, Chandler, AZ, USA) with parallel plates of 25 mm diameter and a 1 mm gap. The experiment was performed at a temperature of 25 °C and at shear rates ranging from 0 to 100 s, following a previously established protocol [28]. The mechanical strength of the gels was assessed using a Stable Micro System TA. XT Plus texture analyzer from Surrey, UK. Cylindrical gel samples measuring 10 mm × 10 mm were molded and refrigerated at approximately 4 °C for 24 h. In the texture profile analysis (TPA) and cyclic compression test, a 25% distance mode with a maximum distance of 2.5 mm was applied, with a test speed of 2 mm/sec. The surface and cross-section morphology of the three different gel compositions were analyzed using a scanning electron microscope (SEM) model TESCAN VEGA3 from Tescan Korea (Seoul, Republic of Korea). Prior to SEM analysis, all samples were lyophilized and coated with platinum sputtering.

### 4.4. 3D (bio) Printing and Four-Axis Printing

For 3D printing, a specialized 3D bioprinting system from SeoulTech (Seoul, Republic of Korea) was implemented. As described in our previous work, the complex 3D structure was designed using Solid Works (Dassault Systems SolidWorks Corp., Waltham, MA, USA), and the G-codes for the STL files were generated using slicing software (Simplify 3D version 4.0, USA) [29,30]. The hydrogel (4 mL) was loaded into the needle-attached plastic syringe (5 mL, Musashi Engineering Inc. (Seongnam, Republic of Korea) (22 gauge). The software was used to adjust the Z-axis (syringe holder), X-axis (stage), and Y-axis (stage) to position the syringe needle close to the substrate-bearing stage. 3D printing utilized a printing speed of 70 mm/min with a pressure of 90 kPa at room temperature (25 °C).

In four-axis printing, the speeds of the X, Y, and Z axes were programmed using G-codes and managed through the 3D printer application on the computer. The rotation of the fourth axis was controlled by a motor operating at 4 rpm. A plastic tube was attached to the rotating cylindrical polypropylene shaft of the motor for the four-axis printing [31].

### 4.5. In Vitro Cytocompatibility Test

The cytotoxicity of the hydrogels was assessed in vitro using the MTT assay. The MTT assay was conducted over a period of three days on the gel samples. For the extraction MTT, 1 mg of each gel was incubated in 1 mL of medium for 48 h. Prior to adding the extract samples to the wells, MC3T3-E1 cells (1 × 10^3^ cells per well) were seeded on a 96-well plate and cultured under standard conditions for 24 h. The following day, the culture medium was replaced with 100 µL of fresh medium, and then 10 µL of each sample extract was added to the wells for the MTT assay. The MTT assay was performed following a previous protocol, and the optical density (OD) values were measured at 570 nm. The relative cell viability, normalized to the control, was reported as a percentage [25].

For the in vitro cell culture experiments, 3D bioprinted samples were prepared using the MC3T3-E1 cells (3 million/mL, passage 13). The cells were cultured in α-Minimum essential media (MEM) supplemented with 10% fetal bovine serum and penicillin-streptomycin (100 units/mL) and maintained in a 37 °C incubator with 5% CO_2_. The in vitro cell culture study was conducted for a duration of 3 days, and observations were made on days 0 and 3. Live/dead staining was performed using the MAX-View™ Live/Dead Cell Staining Kit. This staining method employs fluorescent calcein-AM (green) to indicate intracellular esterase activity and red-fluorescent ethidium homodimer-1 to confirm the loss of plasma membrane integrity. Images of the live and dead cells were captured after the addition of 1.2 µL of 2 mM ethidium homodimer-1 (EthD-1) and 0.3 µL of 4 mM calcein AM to 600 µL of phosphate-buffered saline (PBS). Following the addition of these agents, the plate was incubated in darkness for 30 min. The live and dead cells were visualized using different filters on a fluorescence microscope (Leica DMLB, Wetzlar, Germany) and combined using LAS-X Leica Microsystems software. Additionally, samples were stained with DAPI (4′,6-diamidino-2-phenylindole) for the nucleus (blue) and rhodamine-phalloidin for F-actin (red) to observe cell growth and proliferation [28].

### 4.6. Statistical Analysis

All the experimental data were provided as mean ± standard deviation (S.D.), and the data were statistically compared using Origin Pro 9’s ONE-WAY ANOVA. Results differences were considered statistically significant when *p* ≤ 0.05 (*) and *p* ≤ 0.01 (**).

## Figures and Tables

**Table 1:** Different compositions of gels and their codes.

SI No.	Sample Composition	Sample Code
1	0.3 g HA + 1.0 g Gelatin + 5 mg Genipin + 20 mL DW	Gel 1
2	0.3 g HA + 1.2 g Gelatin + 5 mg Genipin + 20 mL DW	Gel 2
3	0.3 g HA + 1.5 g Gelatin + 5 mg Genipin + 20 mL DW	Gel 3

## Data Availability

The data of this study is available from the corresponding author upon request.

## Supplementary Materials

The following supporting information can be downloaded at: https://www.mdpi.com/article/10.3390/gels9080601/s1, Figure S1. Swelling of different gelatin-HA hydrogel at 37 °C in pH 7.4 phosphate buffer solution (PBS); Figure S2. Degradation study of different gelatin-HA hydrogels at pH 2.5 and pH 9 PBS in 37 °C for 48 h; Figure S3. Degradation study of different gelatin-HA hydrogels at 37 °C and pH 2.5 in PBS after 5 days, where (a) Gel 1; (b) Gel 2; (c) Gel 3; Figure S4. Adhesiveness test of the printed gelatin-HA gel scaffolds by inverting the glass substrate, where (a) star, (b) tube; Video S1: Supporting video file 1-4axis 3D printing; Video S2: Supporting video file 1-Gelatin-HA gel; Video S3: Supporting video file 2-flexibility test; Video S4: Supporting video file 2-Gelatin-HA gel.
