# Supplementary material for: Study on Bioresponsive Gelatin-Hyaluronic Acid-Genipin Hydrogel for High Cell-Density 3D Bioprinting
Source: Gels. 2023 Jul 26;9(8):601. doi: 10.3390/gels9080601 (PMC10453927; doi:10.3390/gels9080601)
Supplement: Supplementary file 1 [file gels-09-00601-s001.zip › supplementary.pdf]

Study on Bioresponsive Gelatin-Hyaluronic Acid-Genipin Hydrogel for High Cell-Density 3D Bioprinting

Mst Rita Khatun [1], Amitava Bhattacharyya [1,2,3], Maral Gunbayar [1], Minsik Jung [1] and Insup Noh [1,3,*]

1 Department of Chemical and Biomolecular Engineering, Seoul National University of Science and Technology, Seoul 01811, Republic of Korea; mstrita931215@seoultech.ac.kr (M.R.K.); amitbha1912@seoultech.ac.kr (A.B.); marlagunbayer@naver.com (M.G.); minsik0217@naver.com (M.J.)
2 Functional, Innovative and Smart Textiles, PSG Institute of Advanced Studies, Coimbatore 641004, India
3 Convergence Institute of Biomedical Engineering and Biomaterials, Seoul National University of Science and Technology, Seoul 01811, Republic of Korea
* Correspondence: insup@seoultech.ac.kr

Scheduled to be submitted to *gels*.

July 2023

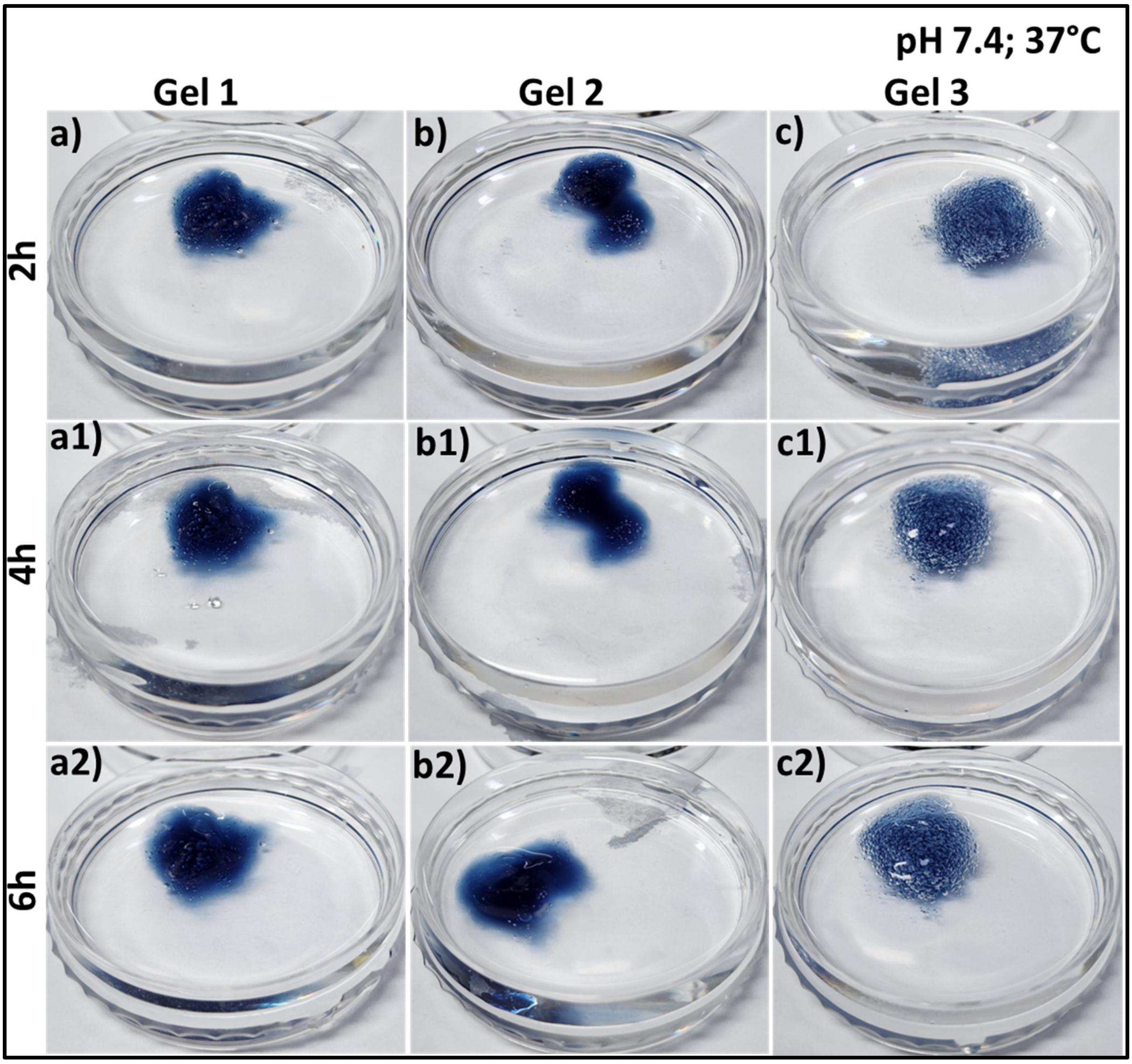

Supporting Figure S1. Swelling of different gelatin-HA hydrogel at 37°C in pH 7.4 phosphate buffer solution (PBS).

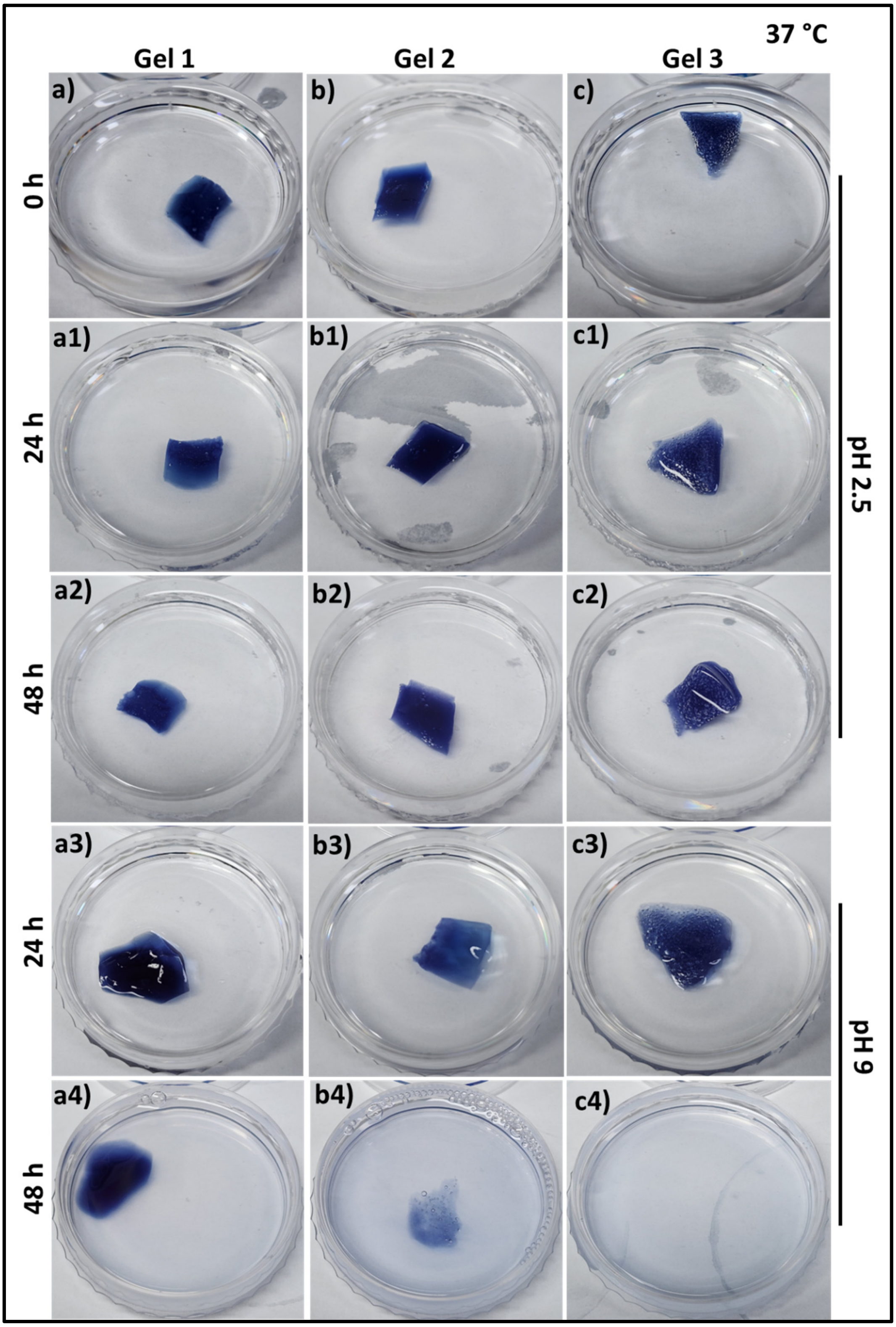

Supporting Figure S2. Degradation study of different gelatin-HA hydrogels at pH 2.5 and pH 9 PBS in 37°C for 48 hours.

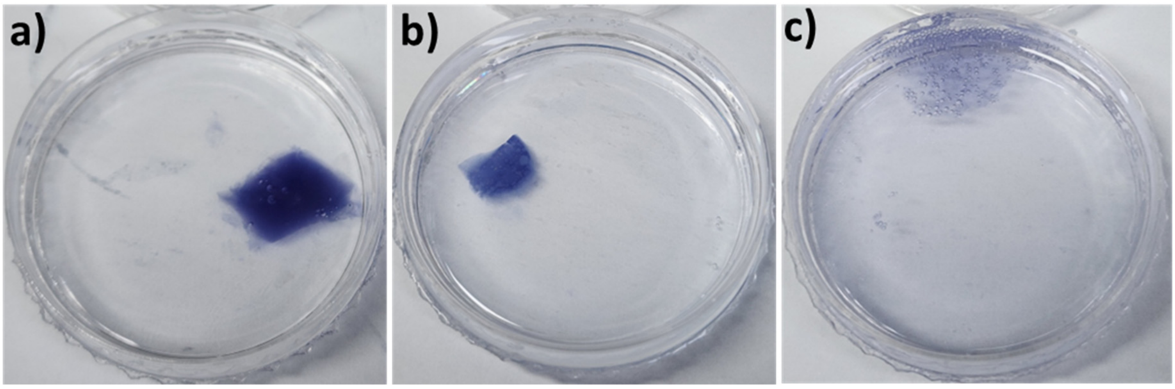

Supporting Figure S3. Degradation study of different gelatin-HA hydrogels at 37°C and pH 2.5 in PBS after 5 days, where (a) Gel 1; (b) Gel 2; (c) Gel 3.

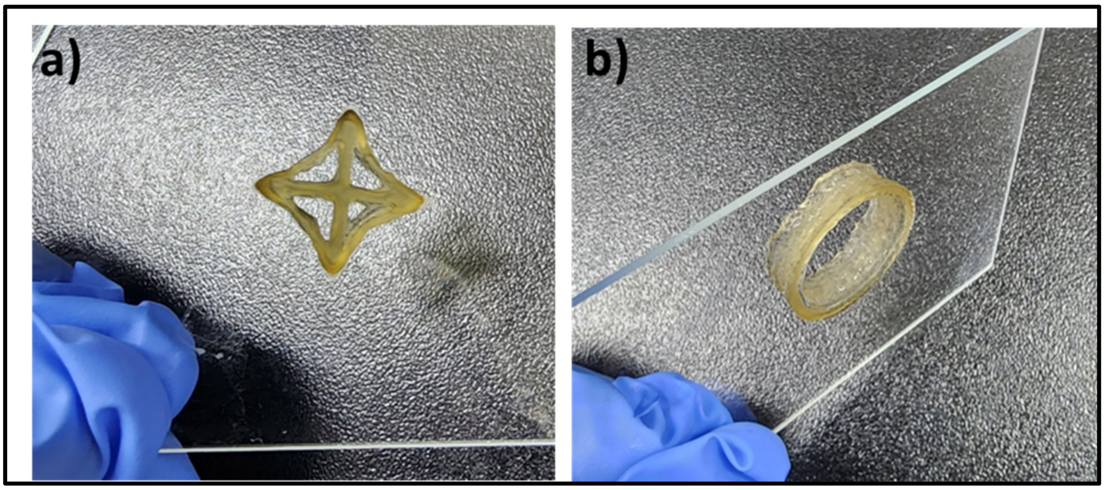

Supporting Figure S4. Adhesiveness test of the printed gelatin-HA gel scaffolds by inverting the glass substrate, where (a) star, (b) tube.
